# Tétanos compliqué de septicémie sur hémoglobinopathie majeure

**DOI:** 10.11604/pamj.2014.19.390.5901

**Published:** 2014-12-18

**Authors:** Savadogo Mamoudou, Lionnet François, Ana Canestri, Guillaume Bellaud

**Affiliations:** 1 Infectious Diseases Department, Yalgado Ouedraogo University Hospital, Ouagadougou, Burkina Faso; 2 Internal Medicine Department, Tenon Hospital Paris, France; 3 Infectious and Tropical Diseases Department, Tenon Hospital Paris, France

**Keywords:** Tétanos, septicémie, hémoglobinopathie, Tetanos, septicemia, hemoglobinopathy

## Abstract

Rapporter un cas de tétanos compliqué de septicémie chez un enfant souffrant d'une hémoglobinopathie majeure. Enfant de 9 ans drépanocytaire (SC), non vacciné contre le tétanos, a été admis au CHU YO pour raideur de la nuque, difficulté à l'ouverture de la bouche et hyperthermie. L'examen à son admission notait un syndrome infectieux avec une hyperthermie (température à 39°1C), Pouls = 100/mn, Fréquence cardiaque = 100batt/mn, fréquence respiratoire = 30cycles/mn, poids =22Kg, un trismus, une contracture des muscles para vertébraux, des paroxysmes toniques à la palpation (stade II de Mollaret), un syndrome méningé. L'examen n'avait pas retrouvé une porte d'entrée. La ponction lombaire avait ramené un liquide céphalo rachidien clair; l'hémogramme avait montré une hyperleucocytose à 10 200/mm^3^, et l'hémoculture a permis d'isoler Staphylococcus aureus. Sous antibiothérapie et sous sédatifs, l’évolution a été favorable. Les porteurs d'hémoglobinopathie majeure sont plus exposés aux infections. La physiopathologie de ces infections s'explique par l'immunodépression et les troubles de la phagocytose. Un dépistage précoce de ces hémoglobinopathies, un bon suivi et une bonne couverture vaccinale des enfants drépanocytaires sont essentiels pour prévenir le tétanos et ses complications.

## Introduction

Chaque année, ce sont 300 000 enfants drépanocytaires qui naissent dans le monde et la majorité provient de l'Afrique [1]. Le Burkina Faso est situé dans la “ceinture sicklémique” de LEHMANN où La drépanocytose constitue un problème de santé publique. C'est une maladie génétique héréditaire du sang où l'hémoglobine normale A est remplacé par l'hémoglobine S. La prévalence de la forme SC représente environ 30% des cas de drépanocytose majeure en Afrique. Ce taux varie entre 52,5% et 62% au Burkina Faso où elle est une cause importante de morbidité et de mortalité, particulièrement chez l′enfant [2]. Les complications aiguës infectieuses de la maladie s'expliquent par l'exclusion fonctionnelle de la rate et l'existence d'infarctus viscéraux où peuvent se multiplier les bactéries responsables des infections [3]. Aussi les patients drépanocytaires sont polytransfusés avec un risque élevé de transmission de maladies virales comme l'infection à VIH et l'hépatite virale B. A notre connaissance, peu d’étude se sont intéressée au Tétanos chez le drépanocytaire. Nous rapportons un cas chez un enfant.

## Patient et observation

Enfant de 9 ans drépanocytaire (SC) connu, non vacciné contre le tétanos, a été admis le 26/06/2014 pour difficulté à l'ouverture de la bouche +raideur de la nuque et fièvre. Cette symptomatologie l'avait amenée en consultation dans une structure sanitaire où un traitement lui avait été administré sans succès. Référé au Centre hospitalier universitaire Yalgado Ouédraogo(CHU YO), l'examen a noté à son admission, un syndrome infectieux avec une température à 39°1C, une fréquence cardiaque = 100/mn, une fréquence respiratoire = 30cycles/mn, un poids = 22 Kg, un trismus, une contracture des muscles para vertébraux, des paroxysmes toniques à la palpation (stade II de Mollaret), un syndrome méningé. L'examen n'avait pas retrouvé une porte d'entrée. La numération formule sanguine(NFS) montrait une hyperleucocytose à 10 200/mm^3^; un taux d'hémoglobine =10g/dl; TCMH = 23,7pg; CCMH = 35,4g/dl, VGM = 67fl, plaquettes = 150000/mm^3^. L'ionogramme montrait une calcémie = 2,82 mmol/l, la créatininémie = 59 micromol/l, les protides = 84g/l, la glycémie = 5,7 micromol/l. Une hémoculture demandée devant le syndrome infectieux avait permis d'isoler Staphylococcus aureus. Mis sous traitement à base de Diazépam 2ampx3/J, de phénobarbital 40mgx2/j Péni G 1millionx2/J, amoxilline + acide clavulanique 500mgx3/J, l’évolution a été favorable.

## Discussion

Les patients drépanocytaires sont plus exposés aux infections dont les plus fréquentes sont les septicémies, les pneumopathies, les pyélonéphrites, et les ostéomyélites [4]. La physiopathologie de ces infections s'explique par plusieurs mécanismes soutenus par l'immunodépression cellulaire et les troubles de la phagocytose. Si le portage de l'hémoglobine S et/ou C semble favoriser l'acquisition d'une immunité contre le paludisme [5], il n'en est pas de même pour le Tétanos, comme constaté par AKPEDE au Nigéria où un enfant drépanocytaire vacciné contre le tétanos sans rappel, est décédé de la maladie [6]. Notre patient n’était pas vacciné contre le Tétanos. Il est déplorable, que le tétanos sévisse au 21ème siècle, malgré l'existence d'un vaccin antitétanique efficace administré gratuitement aux enfants dans le cadre du programme élargi de vaccination (PEV) [7]. A neuf ans, âge de notre patient, ce sont habituellement les crises vaso occlusives osseuses hyperalgiques qui dominent les manifestations cliniques du drépanocytaire; les complications infectieuses sont prédominantes avant sept ans. Ces complications infectieuses sont particulièrement graves et peuvent engager le pronostic vital [8]. Le tétanos était une forme compliquée dont la porte d'entrée n'avait pas été retrouvée, comme chez 20% des cas des séries africaines et européennes [8]. Il était une forme généralisée et modérée (stade II de Mollaret) comme rencontrée dans la plupart des séries africaines. Le Tétanos était compliqué de septicémie à Staphylococcus aureus. Au Kenya Thomas et al avaient noté que Staphylococcus aureus faisait parti des cinq germes responsables de bactériémie dans sa série [9]. Ces septicémies sont souvent dues au portage ORL du staphylocoque. Elles peuvent faire suite à une thrombophlébite adjacente, d'où peuvent partir des emboles septiques responsables de bactériémie. Mais elles sont souvent liées aux voies d'abord veineuses. L’évolution de la maladie a été favorable. L′arrivée des antibiotiques modernes permet de combattre efficacement les infections qui restent néanmoins très dangereuses chez le drépanocytaire. L'amélioration de la prise en charge pédiatrique permet à la très grande majorité des enfants d'atteindre l’âge adulte dans les pays occidentaux [10].

## Conclusion

La drépanocytose est une maladie grave dont l’évolution peut être émaillée de complications infectieuses. Une bonne couverture vaccinale, associée à une antibioprophylaxie, contribueront à réduire ces complications. En cas de septicémie, la rapidité de la mise sous traitement et la prise en compte de la sensibilité aux antibiotiques, sont des éléments fondamentaux de la stratégie thérapeutique. Aussi le diagnostic précoce de la drépanocytose par isoélectrofocalisation (IEF) et la chromatographie liquide à haute performance (CLHP) permet un bon suivi vaccinal nécessaire pour prévenir le Tétanos et ses complications.
